# The application of esketamine in cancer-related depressive states: attention from mechanisms to clinical translation

**DOI:** 10.3389/fpsyt.2026.1781915

**Published:** 2026-07-30

**Authors:** Liangju Chen, Yonghua Chen, Ying Zhao, Tingting Feng, Xiaoyu Song, Fangmei Xie, Jian Shen, Jinhua He, Jinquan Guo, Lin Zhong

**Affiliations:** 1 Central Laboratory, The Affiliated Panyu Central Hospital, Guangzhou Medical University, Guangzhou, China; 2 School of Life Science, South China Normal University, Guangzhou, China; 3 Emergency Medicine Department, The Affiliated Panyu Central Hospital, Guangzhou Medical University, Guangzhou, China; 4 Department of Breast Surgery, The Affiliated Panyu Central Hospital, Guangzhou Medical University, Guangzhou, China; 5 Department of Anesthesiology, The Affiliated Panyu Central Hospital, Guangzhou Medical University, Guangzhou, China

**Keywords:** cancer, clinical translation, depressive state, esketamine, mechanism

## Abstract

**Objectives:**

This article reviews the clinical status and treatment challenges of cancer-related depressive states, clarifies the mechanism of action of esketamine—a novel rapid-acting antidepressant—compares its efficacy with traditional drugs, evaluates safety and tolerability, and summarizes clinical evidence to support clinical practice and research.

**Methods:**

By searching databases such as PubMed, Web of Science, and Cochrane Library, We conducted a structured review of the available literature on the mechanism of esketamine in the treatment of cancer-related depressive states in recent years, including mechanism studies, clinical trials, and review articles. We focused on its regulation of NMDA receptors, the impact on BDNF expression, synaptic plasticity, and neural circuit remodeling, and compared its efficacy and safety with traditional antidepressants.

**Results:**

Esketamine exerts rapid antidepressant effects via multiple mechanisms: NMDA receptor antagonism, downstream signaling modulation, BDNF upregulation, synaptic plasticity enhancement, and neural circuit remodeling. Available studies suggest that esketamine may provide rapid antidepressant effects in oncology populations, particularly in perioperative settings. Common adverse events—such as transient dissociation and elevated blood pressure—are generally controllable.

**Conclusions:**

Esketamine, as a rapidly acting antidepressant, has a plausible mechanistic rationale and potential clinical value in the treatment of cancer-related depressive states. However, its long-term safety, optimal dosing regimens, and individualized application in different cancer patients still require further investigation. In the future, the integration of multi-omics technologies and clinical translational research should be promoted to advance the precision and standardization of its application in cancer-related depressive states treatment.

## 1 Introduction

Cancer remains a critical global health challenge, threatening patient survival while concurrently imposing substantial psychological distress, among which depression is one of the most prevalent comorbid conditions. The pooled prevalence of depressive symptoms among cancer patients during and after treatment ranges from 8% to 24% (1). In a meta-analysis (2), the prevalence of comorbid depression among cancer patients in mainland China was 28.09% (diagnostic interviews) and 44.63% (self-reports). cancer-related depressive states not only impairs patients’ quality of life, but also undermines treatment adherence, worsens prognosis, and increases the risk of suicide. However, the recognition and management of cancer-related depressive states remain challenging. Conventional antidepressants are characterized by delayed onset of action, and a substantial proportion of patients exhibit inadequate therapeutic response. In recent years, esketamine—a novel fast-acting antidepressant—has emerged as a promising therapeutic option for cancer-related depressive states.

**Figure 1:**
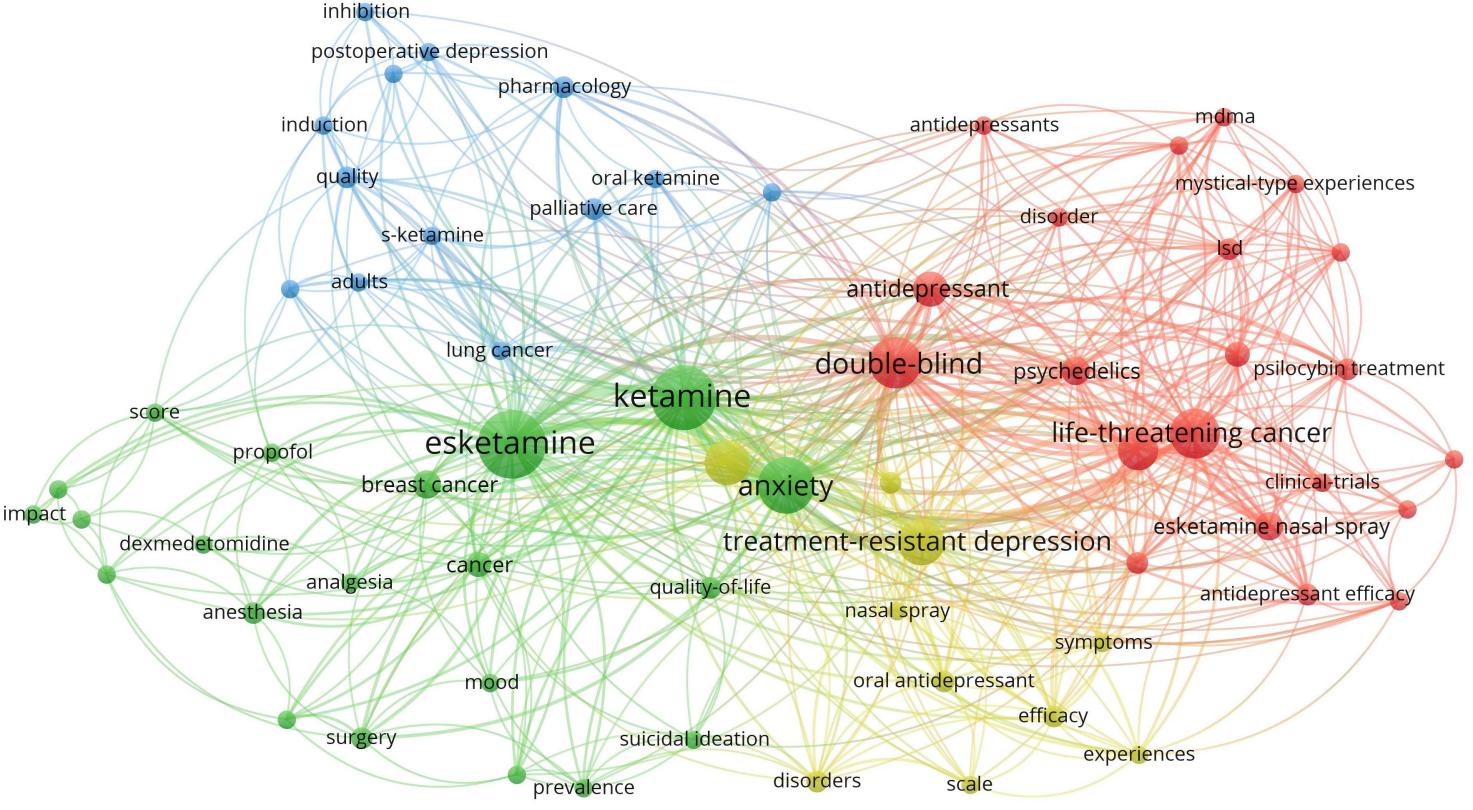
Keyword knowledge graph of esketamine application research in cancer-related depressive states.

A keyword knowledge map of esketamine in cancer-related depressive states was created from 40 Web of Science articles using the terms “esketamine,” “cancer,” and “depressive state.” High-frequency keywords were defined as those appearing more than twice. Key research areas include esketamine’s use in refractory and perioperative depression, administration routes, efficacy, and interactions with other psychotropic agents. However, studies on its mechanism of action remain limited (**Table 1**; **Figure 1**).Therefore,This review outlines the clinical burden and treatment challenges of cancer-related depressive states and assesses esketamine as a novel rapid-acting antidepressant. It examines esketamine’s mechanisms—NMDA receptor antagonism, downstream signaling modulation, BDNF upregulation, synaptic plasticity, neural circuit remodeling, and neuroinflammatory regulation—highlighting its neurobiological basis. The drug’s advantages over conventional antidepressants, including faster onset and efficacy in treatment-resistant cases, are presented. Key safety aspects include common adverse effects, management strategies, use in specific cancer populations, and long-term risks with monitoring. A synthesis of clinical evidence evaluates esketamine’s effectiveness across cancer types. Finally, future directions such as biomarker-guided therapy, individualized treatment, and new delivery methods are discussed, along with current limitations. This review aims to support clinical application and inform future research on esketamine in cancer-related depressive states.

**Table 1:** Cluster of keywords in the Esketamine Application Research in Cancer-related depressive states.

Cluster	Color	Keywords
1	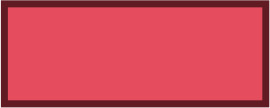	Focusing on clinical research and psychiatric drug synergy, keywords include clinical trials, double-blind studies, psychedelics, psilocybin treatment, life-threatening cancer, etc., reflecting the clinical trial design of esketamine in the treatment of cancer-related depressive states and its associated research with other psychiatric drugs (such as psychedelics).
2	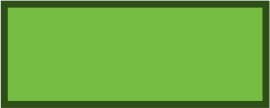	Focusing on the fields of anesthesia and perioperative care, with keywords including propofol, dexmedetomidine, anesthesia, surgery, etc., highlighting the application of esketamine in the anesthesia and perioperative management of cancer patients, while also relating to postoperative depression, suggesting its potential value in intervening in post-cancer surgery depression.
3	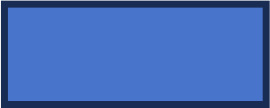	Focusing on pharmacology and basic research on postoperative depression, with keywords such as pharmacology, postoperative depression, inhibition, etc., exploring the pharmacological mechanisms of esketamine and its relationship with postoperative depression.
4	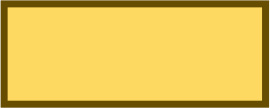	Focusing on the treatment of mood disorders (depression, anxiety), the core keywords include anxiety, treatment-resistant depression, and nasal spray (the method of administering esketamine), reflecting the exploration of esketamine for treating depression and anxiety in cancer patients, especially in cases that are resistant to conventional treatments.

## 2 Methods

This review aimed to systematically summarize and critically evaluate the existing evidence on the application of esketamine in cancer-related depressive states, with a focus on its mechanisms of action, clinical efficacy, safety, and future directions. This review was conducted following the Preferred Reporting Items for Systematic Reviews and Meta-Analyses 2020 statement.

### 2.1 Literature search strategy

A comprehensive literature search was conducted in the following electronic databases: PubMed, Web of Science. The search was limited to articles published in English from inception until October 2025. The following keywords were used in various combinations: “esketamine” “cancer” “depressive state” “depression” “cancer-related depressive states”.

### 2.2 Inclusion and exclusion criteria

Studies were included if they met the following criteria: (1) original research articles, including clinical trials, cohort studies, case-control studies, and case series, or high-quality systematic reviews with meta-analyses; (2) study population: patients with cancer, regardless of cancer type or stage, with or without a formal diagnosis of major depressive disorder, but reporting depressive symptoms or depression-related outcomes; (3) intervention: esketamine administered by any route at any dose or frequency; (4) outcomes: at least one measure of depressive symptom severity, safety/tolerability data, or mechanistic data relevant to cancer-related depressive states; (5) full-text availability via institutional access.

Exclusion criteria were: (1) non-English articles; (2) conference abstracts, editorials, letters without original data, or study protocols without results; (3) animal studies or *in vitro* studies without human clinical data; (4) articles without full text accessible even after institutional library request.

### 2.3 Screening and selection process

**Figure 2:**
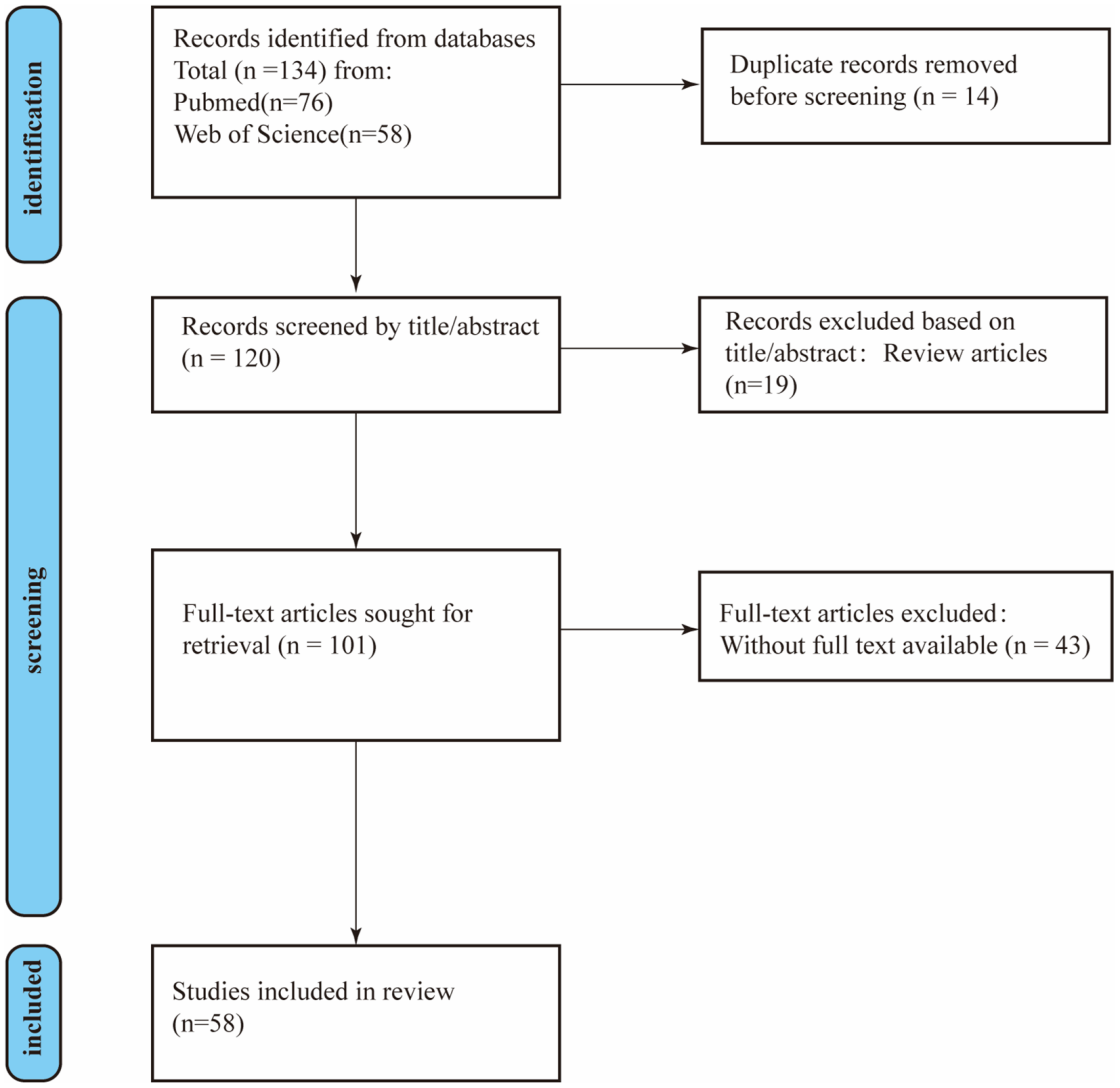
Flow diagram of the article-selection process.

Two authors independently screened titles, abstracts, and full texts based on the predefined inclusion/exclusion criteria. Disagreements were resolved by discussion or consultation with a third reviewer. The detailed review process, including reasons for exclusion at each stage, is shown in **Figure 2**.

## 3 Results

### 3.1 Esketamine’s mechanism of action: unveiling the neurobiological foundations

#### 3.1.1 NMDA receptor modulation and downstream signaling pathways: core molecular mechanisms of esketamine action

**Figure 3:**
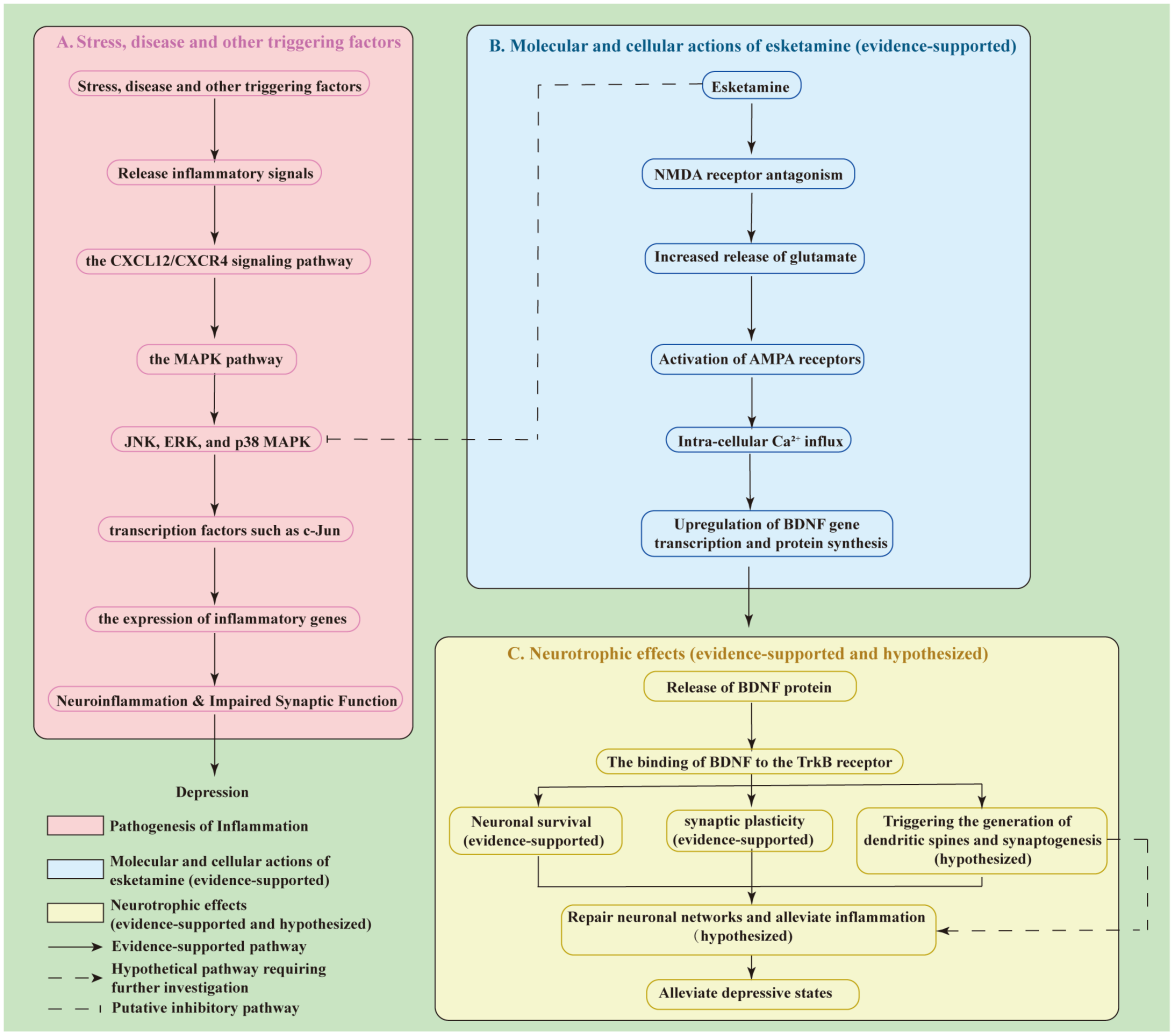
Mechanistic flowchart of Esketamine’s effects on cancer-related depressive states. Solid arrows indicate evidence-supported pathways. Dashed arrows indicate hypothetical pathways requiring further investigation. Dashed lines ending with a bar (⊣) indicate inhibitory pathways.

As a non-selective NMDA receptor antagonist, esketamine exerts its antidepressant effects primarily through modulation of NMDA receptor activity and downstream signaling pathways (**Figure 3**). The NMDA receptor, a major glutamate receptor subtype, is widely expressed in the cerebral cortex and hippocampus, where it regulates synaptic transmission, plasticity, and neuronal excitability. Esketamine selectively blocks NMDA receptors on GABAergic interneurons, suppressing their activity and disinhibiting pyramidal neurons, leading to a transient glutamate surge. This rapidly activates AMPA receptors and triggers downstream signaling pathways, including BDNF release and mTOR activation (3, 4). The study by Duan et al. (5) provides a clear example of esketamine’s modulation of downstream signaling pathways. In the cancer-induced bone pain (CIBP) model, spinal CXCL12/CXCR4 activation triggers the MAPK pathway (JNK, ERK, p38 MAPK) and c-Jun. Esketamine reduces CIBP-related pain and neuronal sensitization by inhibiting the JNK-c-Jun axis. Intrathecal SP600125 or esketamine both alleviate TCI-induced pain and lower p-JNK and p-c-Jun levels in microglia. These results suggest esketamine exerts analgesic and antidepressant effects by modulating neuroinflammation and neuronal function. NMDA receptor antagonism thus drives neurobiological changes that enhance synaptic plasticity and neural circuit activity, supporting its rapid antidepressant action.

#### 3.1.2 BDNF expression regulation: a central mechanism underlying esketamine-mediated neural regeneration

Esketamine’s regulation of brain-derived neurotrophic factor (BDNF) expression represents another key mechanism underlying its antidepressant effects (**Figure 3**). BDNF is a neurotrophic factor essential for neural plasticity, synaptic transmission, and neuronal survival, and is implicated in the pathophysiology of depression. Its expression is significantly reduced in key brain regions—including the prefrontal cortex and hippocampus—of individuals with depression. Studies have demonstrated (6) that traditional antidepressants enhance neuroplasticity and neurotrophic mechanisms through upregulation of BDNF in both peripheral tissues and key brain regions. Esketamine produces rapid and sustained antidepressant effects by activating glutamate-related pathways, accompanied by BDNF-dependent neurotrophic actions. For instance, Jiang et al. (7) demonstrated that a low dose of esketamine increases serum levels of 5-hydroxytryptamine, dopamine, and BDNF in patients with postpartum depression and leads to a short-term reduction in Edinburgh Postnatal Depression Scale (EPDS) scores, resulting in improved depressive symptoms. Specifically, following administration of 0.25 mg/kg esketamine, serum BDNF levels significantly rose from 3.07 ± 0.89 ng/mL pre-administration to 5.45 ± 0.81 ng/mL on day 3 post-treatment (*P* < 0.001). However, it should be noted that Pardossi et al. (6) highlighted inconsistencies in clinical findings: while most studies report increased plasma BDNF levels following intravenous ketamine administration, others present conflicting results. Furthermore, research specifically investigating BDNF in the context of esketamine remains limited. Therefore, larger-scale studies are required to further elucidate the impact of esketamine on BDNF, including investigations of intranasal esketamine, to achieve a more comprehensive understanding of its mechanism of action.

#### 3.1.3 Synaptic plasticity and neural circuit remodeling: neurobiological mechanisms of esketamine

The impact of esketamine on synaptic plasticity and neural circuit remodeling is also a crucial aspect of its antidepressant effect (**Figure 3**). Synaptic plasticity, defined as the ability of synaptic connections between neurons to undergo strengthening or weakening, serves as a fundamental basis for learning, memory, and adaptation to environmental changes. Esketamine rapidly enhances synaptic plasticity, leading to the restoration of neural circuits impaired by chronic depression (8). For instance, Woojin Kim et al. (9) demonstrated that chronic pain induces maladaptive plasticity in the sensory nervous system, and neural circuit remodeling in the cerebral cortex may contribute to the persistence of chronic pain symptoms. Esketamine may exert its therapeutic effects by reversing these maladaptive changes. Furthermore, esketamine may modulate neural circuit remodeling—the process involving the formation of new synaptic connections and the elimination of existing ones—thereby supporting functional reorganization of brain networks. T. Picart et al. (10) demonstrated that neurons and glioma cells engage in complex bidirectional interactions, with glioma cells forming a highly interconnected network via tumor microtubules. These findings provide a theoretical framework for exploring whether esketamine may influence neural circuit remodeling, although direct evidence in cancer patients is currently lacking.

#### 3.1.4 Inflammatory cytokine network modulation: esketamine’s regulation of the immune microenvironment in cancer-related depressive states

The mechanism underlying esketamine’s regulation of the inflammatory response in cancer-related depressive states is complex and remains an active area of investigation. Cancer patients frequently exhibit chronic inflammation, which is closely associated with the onset and progression of depressive symptoms (11). Andersen et al. (12) indicated that depressive symptoms in patients with non-small cell lung cancer (NSCLC) are significantly associated with systemic inflammatory biomarkers, and individuals with severe depressive symptoms are more likely to exhibit unfavorable prognostic inflammatory profiles. These findings suggest that esketamine may alleviate cancer-related depressive states by modulating inflammatory responses. Marinovic et al. (13) showed that depression involves chronic activation of pro-inflammatory cytokines and HPA axis dysregulation, and that interventions like mindfulness meditation can reduce inflammation by lowering cytokine and cortisol levels—potentially improving cancer outcomes. Given that esketamine exerts both anti-inflammatory effects and direct central antidepressant actions, a key question arises: does esketamine improve depressive symptoms indirectly by affecting cancer progression, or independently of cancer status? Available evidence suggests two parallel mechanisms. On one hand, esketamine reduces pro-inflammatory cytokines and attenuates systemic inflammation, which may indirectly alleviate inflammation-associated depressive symptoms (14). On the other hand, esketamine enhances synaptic plasticity via NMDA/AMPA/BDNF/mTOR pathways, a direct antidepressant mechanism well-validated in non-cancer patients with treatment-resistant depression, indicating that its antidepressant effects can occur independently of changes in cancer status (15).

Currently, no direct evidence distinguishes these two mechanisms, and most existing studies focus on perioperative short-term use with limited long-term follow-up (16). Future studies should examine whether esketamine’s antidepressant action is mediated by suppressing key inflammatory pathways such as IL-6 and TNF-α, and could be designed to differentiate between direct and indirect mechanisms. For example, researchers could compare esketamine’s antidepressant effects in patients with different tumor burden status.

#### 3.1.5 Integrative network of esketamine’s mechanisms: interplay among NMDA receptor modulation, BDNF expression, synaptic plasticity, and neuroinflammation

While the preceding sections have detailed four core mechanisms of esketamine, these processes do not operate in isolation. Rather, they form a complex, interconnected regulatory network that collectively underlies esketamine’s rapid and sustained antidepressant effects.

##### 3.1.5.1 Molecular pathways linking NMDA receptor antagonism to synaptic plasticity remodeling

NMDA receptor antagonism by esketamine triggers a cascade of molecular events culminating in structural and functional synaptic plasticity (3). The transient glutamate surge following GABAergic disinhibition activates postsynaptic AMPA receptors, leading to depolarization and calcium influx. This calcium influx activates multiple downstream signaling cascades. First, the mTORC1 pathway is activated via PI3K-Akt signaling, promoting translation of synaptic proteins including GluA1 and PSD-95, which are essential for dendritic spine formation (17). Second, the CaMKII pathway is engaged, leading to phosphorylation of AMPA receptors and enhanced synaptic transmission (18). Third, the ERK-MAPK pathway is activated, contributing to gene expression changes that support long-term plasticity (18).

##### 3.1.5.2 Bidirectional relationship between neuroinflammation and BDNF expression

A critical upstream-downstream relationship exists between neuroinflammation and BDNF expression. Pro-inflammatory cytokines (IL-6, TNF-α, IL-1β) suppress BDNF transcription through multiple mechanisms: activation of nuclear factor kappa-B(NF-κB) pathway inhibits CREB-mediated BDNF gene expression; p38 MAPK activation reduces BDNF mRNA stability; and inflammatory signaling promotes histone deacetylation at BDNF promoters (19, 20). This represents an upstream regulatory relationship (inflammation → reduced BDNF).

Conversely, BDNF exerts feedback regulation on inflammation. BDNF binding to tropomyosin receptor kinase B (TrkB) receptors on microglia and immune cells activates downstream signaling that suppresses pro-inflammatory cytokine release and promotes alternative (M2) microglial polarization (19). This establishes a negative feedback loop that can perpetuate depressive symptoms when disrupted. Esketamine may interrupt this cycle by rapidly increasing BDNF (3, 19).

##### 3.1.5.3 An integrated hierarchical model

Based on the above analysis, we propose an integrated hierarchical model with feedback regulation (3, 15, 19):

This model illustrates that NMDA receptor antagonism is the upstream trigger, BDNF/mTOR signaling serves as the central hub, and synaptic plasticity changes together with anti-inflammatory effects represent the functional outcomes. The bidirectional relationship between BDNF and neuroinflammation establishes a self-reinforcing loop that may explain both the persistence of depressive symptoms and the sustained therapeutic effects of esketamine (19, 20).

### 3.2 Pharmacological management of cancer-related depressive states: comparative efficacy and recent advances

#### 3.2.1 Efficacy and limitations of conventional antidepressant therapies

Selective serotonin reuptake inhibitors (SSRIs) are first-line pharmacological agents for the treatment of depression, exerting their therapeutic effects through selective inhibition of serotonin reuptake, thereby increasing synaptic serotonin concentrations. While SSRIs have a favorable safety profile overall, they are associated with adverse effects such as sexual dysfunction (21) and gastrointestinal disturbances, which may compromise treatment adherence and lead to premature discontinuation. Despite their broad clinical use, a substantial proportion of patients exhibit inadequate response to SSRI monotherapy.

Serotonin-norepinephrine reuptake inhibitors (SNRIs) produce antidepressant effects by selectively inhibiting the reuptake of serotonin and norepinephrine, thereby enhancing synaptic concentrations of these neurotransmitters. Widely used in the management of major depressive disorder, SNRIs demonstrate potential clinical advantages over SSRIs, particularly in patients with comorbid pain or fatigue. Common adverse effects—such as nausea, abdominal pain, and sedation—are typically mild to moderate and rarely lead to treatment discontinuation (22).Furthermore, SNRIs have established analgesic properties and are increasingly utilized for treating chronic pain conditions, especially those comorbid with depression (23).

Tricyclic antidepressants (TCAs), as early-generation agents in the treatment of depression, have demonstrated modest efficacy in alleviating depressive symptoms and improving overall symptomatology in conditions such as irritable bowel syndrome. However, their clinical use is limited by significant safety concerns. Patients receiving TCAs exhibit a higher risk of treatment discontinuation due to adverse effects, indicating potential challenges with tolerability and adherence (24). In the treatment of adolescent depression, TCAs show limited therapeutic benefit and are characterized by a high number needed to treat (NNT), underscoring their suboptimal efficacy in this population (25).

Noradrenergic and specific serotonergic antidepressants (NaSSAs), such as mirtazapine, exert their antidepressant effects by blocking α2-adrenergic autoreceptors and heteroreceptors, as well as specific 5-HT receptor subtypes, thereby increasing synaptic concentrations of norepinephrine and serotonin. Unlike SSRIs and SNRIs, which primarily act through reuptake inhibition, NaSSAs have a distinct mechanism of action based on receptor antagonism, contributing to a unique adverse effect profile and differential clinical utility. Evidence from clinical studies indicates that the incidence of adverse reactions is significantly higher in patients receiving NaSSAs compared to those on placebo, underscoring notable differences in tolerability that must be considered when selecting appropriate pharmacotherapy (26). Mirtazapine is particularly prescribed for its potent sedative properties, making it a valuable therapeutic option for individuals with depression and comorbid insomnia.

In summary, Traditional antidepressants exhibit limited efficacy, characterized by delayed therapeutic onset and low remission rates. Despite the widespread clinical use of SSRIs and SNRIs, many patients experience meaningful symptom improvement only after several weeks to months, and a substantial proportion fail to achieve full remission (27). Even with sequential or combination pharmacotherapy, a significant number of individuals remain treatment-resistant, underscoring a major limitation in current antidepressant strategies.

Common side effects of traditional antidepressants significantly compromise patient adherence. Adverse drug reactions (ADRs) are strongly associated with treatment discontinuation, as many patients discontinue therapy due to poor tolerability. In individuals with depression, symptoms such as drowsiness and headache are linked to higher dropout rates during SSRI treatment (28). Approximately 38.1% of SSRI users experience at least one adverse effect, with gastrointestinal disturbances being the most prevalent (26.7%). The incidence of side effects is markedly higher among short-term users (<3 months; 55.3%) compared to long-term users (14.3%), suggesting that early treatment phases pose greater tolerability challenges (29). Furthermore, patient-related factors such as pre-existing negative beliefs or misconceptions about antidepressants independently contribute to non-adherence (30). (**Table 2**).

**Table 2:** Integrated Hierarchical Model of Esketamine’s Mechanisms.

Level	Mechanism	Role in the network	References
Level 1 (Trigger)	NMDA receptor antagonism (GABAergic disinhibition)	Upstream initiator	(3)
Level 2 (Amplification)	Glutamate surge **→** AMPA receptor activation	Signal amplification	(18)
Level 3 (Execution)	BDNF release **→** TrkB activation **→** mTOR signaling	Central hub	(3, 17, 19)
Level 4 (Outcome)	Synaptic plasticity remodeling **+** Anti-inflammatory effects	Functional endpoints	(3, 19)
Feedback	BDNF **→** suppression of inflammation; Inflammation **→** suppression of BDNF	Bidirectional regulatory loops	(19, 20)

→ represent a directional signaling cascade, consistently meaning 'leads to', 'activates', or 'promotes' the downstream molecular event in each

#### 3.2.2 The advantages of esketamine over traditional antidepressants

The delayed therapeutic onset of antidepressants targeting the monoamine neurotransmitter system has created a pressing need for faster-acting and more effective treatments. In response, and grounded in the glutamatergic hypothesis of depression, research focus has increasingly shifted toward investigating the pharmacological properties of ketamine (31). Among its derivatives, esketamine demonstrates distinct clinical advantages over conventional antidepressants, particularly in the management of treatment-resistant depression (TRD) and in achieving rapid symptom relief—often within hours to days—offering a critical therapeutic option for patients with severe or acute depressive episodes.

Esketamine’s unique mechanism of action underlies its rapid therapeutic onset. Unlike traditional antidepressants, which primarily modulate monoamine neurotransmitters such as serotonin and norepinephrine and typically require several weeks to months to achieve clinical effects, esketamine acts as an N-methyl-D-aspartate (NMDA) receptor antagonist that rapidly restores glutamatergic neurotransmission. This pharmacological profile enables significant improvement in depressive symptoms within hours following a single administration (32). The speed of symptom relief conferred by esketamine is particularly critical for patients with suicidal ideation or severe functional impairment, offering a vital intervention window in acute depressive episodes (33).

Esketamine has demonstrated significant efficacy in the treatment of TRD. Patients with TRD typically exhibit poor or inadequate responses to conventional antidepressants; however, esketamine effectively alleviates depressive symptoms through its distinct mechanism of action involving NMDA receptor modulation. Clinical studies have consistently shown that esketamine, when used as an adjunct to standard antidepressant therapy, yields significantly higher clinical response and remission rates compared to placebo in patients with TRD (34). These findings underscore its therapeutic value in this difficult-to-treat population. (**Table 3**).

**Table 3:** Comparison of antidepressant medications.

Drug class	Representative agent	Typical dosage	Method of administration	Time to onset	Primary therapeutic effect	Adverse effects	Advantages	Disadvantages	Literature source
SSRIs	Sertraline Escitalopram Paroxetine	Sertraline: 50–200 mg/d Escitalopram: 10–20 mg/d	oral administration	2–4 weeks	Remission rate for mild to moderate depression: 50–65% Improvement in anxiety symptoms	Nausea Sexual dysfunction Hyponatremia (in the elderly)	High safety Good tolerance Controllable drug interactions	Slow onset of action Limited effect on severe depression CYP450 enzyme interactions	(21, 22, 35–41)
SNRIs	Duloxetine Venlafaxine	Duloxetine: 60–120 mg/d Venlafaxine: 75–225 mg/d	oral administration	2–4 weeks	Depression remission rate: 55-70% Significantly improves cancer pain	Nausea Hypertension Constipation	Advantages of pain comorbidity Improving fatigue symptoms Dual regulatory effect	Cardiovascular risk Withdrawal syndrome Obvious gastrointestinal reactions	(22, 23, 42–46)
TCAs	Amitriptyline Nortriptyline	Amitriptyline: 25–150 mg/d Nortriptyline: 25–100 mg/d	oral administration	2–4 weeks	Remission rate of treatment-resistant depression: 40–60% Reduction in opioid dosage	Dry mouth Constipation Cardiotoxicity	Effective for treatment-resistant depression Low cost Pain relief assistance	Significant side effects High risk of overdose Narrow therapeutic window	(24, 25, 36, 47, 48)
NaSSA	Mirtazapine	15–45 mg/d	oral administration	2–4 weeks	Appetite score ↑ 40% Weight gain ≥ 2 kg Insomnia relief rate 58%	Weight gain (25-30%) Daytime sleepiness Elevated blood sugar	Improve cachexia Promote sleep Take effect relatively quickly	Metabolic side effects Risk of granulocytopenia Use with caution in obese patients	(26, 42, 49)
Esketamine	Esketamine nasal spray	56–84 mg per dose Twice a week	Nasal spray	24–48 hours	Remission rate: 71.8% Pain score decreased by 30-40% Rapid improvement in suicidal ideation	Transient dissociative symptoms Elevated blood pressure (10–15 mmHg) Dizziness	Rapid onset of effect Highly effective for treatment-resistant depression Synergistic analgesic effect Immune regulatory potential	Clinical monitoring for 2 hours is required. Use with caution in patients with cardiovascular diseases. Long-term safety remains to be studied.	(31–34, 50–52)

## 4 Discussion

### 4.1 Clinical implications

#### 4.1.1 The application of esketamine in breast cancer-related depressive states

Esketamine, a rapid-acting antidepressant, has emerged as a promising therapeutic agent for breast cancer-related depressive states. Breast cancer patients are particularly vulnerable to severe depressive symptoms due to the psychological impact of diagnosis, treatment-related side effects, body image disturbances, and persistent fear of recurrence. This comorbid depression not only significantly impairs quality of life and social functioning but may also compromise antitumor immunity through neuroendocrine dysregulation, potentially diminishing treatment efficacy.

Breast cancer patients face multiple psychosocial and physiological stressors, including the psychological impact of diagnosis, treatment-related physical discomfort, body image disturbances, and persistent fears regarding prognosis and recurrence. These factors act synergistically to increase the risk of developing severe depressive symptoms, with prevalence rates substantially higher than those observed in the general population. This comorbid depression not only profoundly impairs quality of life and social functioning but may also disrupt immune regulation via neuroendocrine pathways, potentially compromising treatment tolerance and oncological outcomes.

A growing body of evidence supports the efficacy of esketamine in alleviating postoperative depression in breast cancer patients. Xu et al. (53) demonstrated that a 4-week course of esketamine (0.5 mg/kg) significantly improved depressive symptoms, as reflected by reduced HAMD scores (*P* < 0.01), through inhibition of hippocampal TREK-1 channels (1.8-fold downregulation) and enhanced GABA release (68% increase), highlighting a novel mechanism for targeted intervention. Wei et al. (54) published a prospective randomized double-blind placebo-controlled trial protocol designed to evaluate whether perioperative low-dose esketamine could improve postoperative depressive symptoms in breast cancer patients. The study was also designed to investigate potential neurobiological mechanisms involving BDNF regulation and functional connectivity of the prefrontal cortex. In a randomized controlled trial, Wang et al. (55) found that administering a sub-anesthetic dose of esketamine (0.2 mg/kg) during surgery significantly reduced sub-threshold depressive symptoms on postoperative day one in patients without baseline depression, as measured by PHQ-9 scores (2.19 ± 2.75 vs. 4.03 ± 3.84, *P* = 0.047). Together, these studies suggest that esketamine may have potential prophylactic and therapeutic value in perioperative breast cancer populations.

Collectively, the available evidence suggests that esketamine may improve postoperative depressive symptoms through both neurochemical and neuroplasticity-related mechanisms. However, the current evidence base remains limited by relatively small sample sizes, short follow-up durations, and the inclusion of protocol-based investigations without published outcome data. Therefore, larger multicenter randomized controlled trials are required to confirm its long-term efficacy and safety in breast cancer populations.

#### 4.1.2 The application of esketamine in lung cancer-related depressive states

Lung cancer is one of the most prevalent and life-threatening malignancies worldwide, posing a significant burden on global public health. Patients not only experience debilitating physical symptoms—including persistent cough, hemoptysis, chest pain, and dyspnea—but also endure substantial psychological distress arising from the disease, such as fear of mortality, concerns about deteriorating quality of life, and anxiety over altered family and social roles. These combined physical and emotional challenges place lung cancer patients at high risk for developing depression. Cancer-related depressive states significantly impair patients’ quality of life and negatively impact treatment adherence and clinical outcomes. Moreover, depression is associated with immunosuppression, increased susceptibility to infections, reduced efficacy of surgical, chemotherapeutic, and radiotherapeutic interventions, and may ultimately contribute to shortened survival duration.

Available evidence suggests that esketamine may have potential antidepressant effects in patients with lung cancer. A randomized controlled trial by Gan et al. (56) showed that continuous perioperative infusion of esketamine during thoracoscopic lung cancer surgery—comprising a 0.1 mg/kg loading dose intraoperatively, followed by a maintenance infusion of 0.1 mg/kg/h, and postoperative patient-controlled intravenous analgesia (PCIA) for 48 hours—reduced the incidence of postoperative depression at one month from 11.8% to 1.3% (*P* = 0.018). Furthermore, a study involving 236 patients with both lung cancer and major depressive disorder (MDD) reported that after eight sessions of esketamine (ESK) treatment, depressive and anxiety symptoms were significantly reduced, and cognitive function was notably improved. In the ESK group, markers of nicotine dependence, smoking cravings, and smoking-related respiratory symptoms also showed significant alleviation (57).

Taken together, the currently available lung cancer studies provide preliminary evidence supporting the antidepressant potential of esketamine in both perioperative and non-perioperative settings. Importantly, beneficial effects have been observed not only in postoperative depressive symptoms but also in patients with established major depressive disorder. Nevertheless, differences in treatment routes, intervention duration, and outcome measures limit direct comparison across studies and warrant further standardized investigations.

#### 4.1.3 The application of esketamine in thyroid cancer-related depressive states

Patients with thyroid cancer, particularly those with well-differentiated subtypes such as papillary carcinoma who typically have a favorable prognosis, exhibit a significantly higher prevalence of depression compared to the general population. This elevated risk arises from the interplay of profound psychological distress following cancer diagnosis, treatment-related adverse effects, and the chronic burden of long-term disease management. Key contributing factors include direct physiological consequences of thyroid dysfunction during treatment—such as fatigue and low mood associated with postoperative hypothyroidism, and anxiety or insomnia induced by subclinical thyrotoxicosis during thyroid-stimulating hormone (TSH) suppression therapy; altered body image due to visible neck scars and voice changes following surgery; the psychological impact of lifelong dependence on levothyroxine replacement.Untreated or inadequately managed depressive disorders can substantially impair patients’ quality of life, reduce adherence to treatment regimens, and may potentially serve as an adverse prognostic factor.

A study (58) has demonstrated that intravenous administration of 0.5 mg/kg esketamine during the induction phase of general anesthesia significantly reduces postoperative anxiety and depression scores in patients undergoing thyroid cancer surgery. A single dose of esketamine effectively decreases the incidence of postoperative anxiety and depressive symptoms, providing a theoretical foundation for its potential use in the prophylaxis of postoperative depression in this patient population.

Although these findings suggest promising antidepressant and anxiolytic effects, the current evidence is derived from a single randomized controlled study with relatively short-term follow-up. Additional studies involving larger and more diverse thyroid cancer populations are required before definitive conclusions can be drawn.

#### 4.1.4 The application of esketamine in cervical cancer-related depressive states

Cervical cancer patients are at substantial risk of developing depression throughout the disease trajectory. The initial diagnosis often induces profound psychological distress, including shock, fear of poor prognosis, and disturbances in body image resulting from treatment interventions such as surgery, radiotherapy, and chemotherapy—particularly due to consequences like loss of fertility and sexual dysfunction—along with associated adverse effects. These factors can trigger intense anxiety, persistent sadness, and diminished self-esteem. During treatment and the recovery phase, patients frequently experience depressive symptoms stemming from disease-related stigma, social withdrawal, ongoing concerns about recurrence and metastasis, financial strain, and the broader impact of illness on future life domains, including intimate relationships and family roles. The cumulative burden of these psychosocial stressors positions depression as a critical determinant of patients’ quality of life, treatment adherence, and long-term survival outcomes, underscoring the urgent need for early recognition and the integration of comprehensive psychological interventions in clinical care.

A randomized, double-blind clinical trial demonstrated that sub-anesthetic doses of S-ketamine (esketamine) can effectively alleviate depressive symptoms and reduce pain perception in patients with mild to moderate depression following cervical cancer surgery, at least in the short term. Compared with racemic ketamine, administration of 0.5 mg/kg S-ketamine led to a significant reduction in Hamilton Depression Rating Scale (HAMD-17) scores—a marked mean decrease observed within 1–3 days postoperatively (*P* < 0.05)—as well as lower Visual Analogue Scale (VAS) pain scores. Furthermore, serum levels of brain-derived neurotrophic factor (BDNF) and serotonin increased significantly, suggesting potential involvement of neurochemical mechanisms in the therapeutic effects. Overall, the S-ketamine group exhibited superior outcomes in both depression relief and pain control compared to the control group, providing robust evidence to support its integration into postoperative management strategies (59).

Compared with other cancer-specific studies, this trial included a relatively large sample size and directly evaluated patients with depressive symptoms. However, the observed benefits were primarily short-term, and the long-term sustainability of these antidepressant effects remains unclear. Future studies should further investigate long-term clinical outcomes and optimal dosing strategies.

#### 4.1.5 The application of esketamine in pancreatic cancer-related depressive states

Pancreatic cancer is a highly aggressive malignancy of the digestive tract characterized by an extremely poor prognosis. The diagnosis itself represents a profound psychological shock, and the severe pain, progressive cachexia, and existential burden associated with the disease substantially increase the risk of developing depressive disorders. Pancreaticoduodenectomy, as the primary curative-intent treatment, offers potential for long-term survival; however, the procedure entails substantial surgical trauma, prolonged recovery, and a high incidence of postoperative complications—such as delayed gastric emptying and impaired digestion and absorption—that may lead to permanent physiological alterations. These factors collectively contribute to a marked decline in patients’ quality of life. Persistent physical distress, irreversible functional deficits, and intense anxiety about disease recurrence further serve as critical contributors to the onset or worsening of depression.

Yu et al. (60) published a prospective randomized controlled trial protocol designed to evaluate the effects of postoperative esketamine combined with sufentanil on depression and analgesia in patients undergoing pancreatoduodenectomy. The planned study includes assessments of HAMD-17 scores, pain outcomes, adverse events, and biological markers including BDNF, 5-HT, TNF-α, and IL-6. As outcome data have not yet been published, the efficacy of esketamine in pancreatic cancer-related depressive states remains to be confirmed. Nevertheless, this protocol highlights growing interest in the potential dual antidepressant and analgesic effects of esketamine in highly invasive cancer surgery.

Given that the currently available evidence consists of a study protocol rather than a completed clinical trial, conclusions regarding efficacy should be considered preliminary. Future findings from this trial may provide important insights into the role of esketamine in simultaneously improving postoperative pain and depressive symptoms following major abdominal cancer surgery.

#### 4.1.6 Evidence synthesis and critical appraisal

Across the eight studies summarized in **Table 4**, six were completed clinical investigations and two were published trial protocols. Most completed studies reported beneficial effects of esketamine on depressive symptoms or emotional recovery in oncology populations. However, several limitations should be considered, including heterogeneous assessment tools, relatively small sample sizes, predominantly short-term follow-up, and limited multicenter validation. Overall, the current evidence supports the potential clinical value of esketamine in the management of cancer-related depressive states. Nevertheless, the available evidence base remains limited and heterogeneous. Therefore, large-scale, multicenter randomized controlled trials with standardized outcome measures and longer follow-up periods are required to further clarify its long-term efficacy, safety, and applicability across different cancer populations.

**Table 4:** Characteristics of Clinical Studies Included in the Review.

Author (year)	Cancer type	Study design	Sample size	Intervention	Assessment	Main findings
Xu et al. (53)	Breast cancer	RCT + mechanistic study	54	0.5 mg/kg SC twice weekly	HAMD-17	Improved postoperative depressive symptoms.
Wei et al. (54)	Breast cancer	RCT protocol	Planned 108	0.25 mg/kg vs saline	Depression outcomes	Protocol study.
Wang et al. (55)	Breast cancer	Double-blind RCT	64	0.2 mg/kg IV	PHQ-9	Lower POD1 PHQ-9 score.
Gan et al. (56)	Lung cancer	Placebo-controlled RCT	156	Perioperative IV esketamine	BDI-II	Reduced 1-month depressive symptoms.
Hong et al. (57)	Lung cancer + MDD	Multicenter RCT	236	Intranasal weekly ×8	Depression/anxiety scales	Improved depression, anxiety and cognition.
Bi et al. (58)	Thyroid cancer	Double-blind RCT	80	0.5 mg/kg IV	SDS/SAS	Reduced anxiety and depression.
Wang et al. (59)	Cervical cancer	Double-blind RCT	417	S-ketamine 0.25/0.5 mg/kg	HAMD-17	Improved short-term depression and pain.
Yu et al. (60)	Pancreatic cancer	RCT protocol	Planned 80	Esketamine + sufentanil	HAMD-17	Protocol study.

### 4.2 Study limitations

#### 4.2.1 Limitations associated with esketamine administration

The limitations associated with esketamine in the treatment of cancer-related depressive states represent critical considerations in clinical practice. Common adverse reactions include dissociative symptoms, elevated blood pressure, nausea, vomiting, dizziness, and headache. Dissociative symptoms—characterized by a subjective sense of detachment from the self or surroundings—may lead to patient discomfort and anxiety. A clinical study in patients with TRD (61) demonstrated that esketamine nasal spray, when combined with oral antidepressants, frequently induced adverse effects such as dizziness, dissociative symptoms (depersonalization or derealization), nausea, vomiting, somnolence, and headache, with high incidence rates; some patients discontinued treatment due to these events. To effectively manage these adverse effects, clinicians should implement a comprehensive approach: closely monitor blood pressure and heart rate during administration and promptly address hypertensive episodes; administer antiemetic agents prophylactically to mitigate gastrointestinal symptoms; and reduce infusion rate or dosage while incorporating supportive psychological counseling to educate patients about expected drug effects, thereby minimizing distress related to dissociation.

#### 4.2.2 Variability in esketamine tolerability and implications for individualized treatment strategies

In specific cancer patient populations, such as older adults or those with multiple comorbidities, esketamine tolerability may be significantly reduced, warranting careful risk-benefit assessment. Aging is associated with impaired hepatic and renal function, leading to slower drug metabolism and clearance, increased accumulation of esketamine and its active metabolites, and higher susceptibility to adverse effects. Patients with cardiovascular conditions, respiratory diseases, or neurological disorders face elevated risks. For instance, patients with uncontrolled hypertension may develop malignant hypertension following medication administration.

Furthermore, anticancer therapies—such as chemotherapy-induced bone marrow suppression or liver injury—can impair metabolic and excretory pathways, increasing systemic exposure and toxicity of esketamine and its metabolites. Prior to initiating esketamine therapy for cancer-related depressive states or refractory cancer pain, a comprehensive and individualized patient evaluation must be conducted, encompassing key factors such as age, comorbid conditions (particularly cardiovascular, respiratory, neurological, psychiatric, and hepatic/renal disorders), tumor type and stage, and current or prior anticancer treatments—including their potential impact on organ function.

#### 4.2.3 Core methodological limitations of the included studies

Beyond the adverse effects and tolerability issues discussed above, the studies included in this review have several core methodological deficiencies that warrant careful consideration when interpreting the findings.

##### 4.2.3.1 Functional unblinding and expectancy bias

The dissociative effects of esketamine pose a significant challenge to maintaining blinding integrity in clinical trials. Since patients in the active treatment group are more likely to discern that they are receiving the active drug rather than placebo, this “functional unblinding” may artificially inflate treatment effect estimates. A cross-sectional analysis of 40 ketamine/esketamine clinical trials by Swieczkowski et al (62). demonstrated that most trials failed to adequately mask the perceptual effects of ketamine, potentially compromising the objectivity of outcome assessments. Fountoulakis et al. (63) conducted a meta-analysis identifying functional unblinding as a potential source of overestimation of esketamine’s antidepressant efficacy. Among the cancer-related depressive states studies included in this review, few reported the use of active placebos to control for dissociative effects, limiting the reliability of efficacy estimates.

##### 4.2.3.2 Lack of long-term safety and efficacy data

Most esketamine clinical trials have relatively short follow-up periods (typically 4 weeks), resulting in an inadequate understanding of its long-term safety profile. Real-world pharmacovigilance data (64) have identified signals for substance abuse (ROR = 6.82) and interstitial cystitis (ROR = 12.15) associated with esketamine, risks that are difficult to fully assess in short-term trials. In cancer patients who may already be receiving long-term opioid therapy, the abuse potential and long-term tolerability of esketamine require further investigation.

##### 4.2.3.3 Heterogeneity in study populations and outcome measures

The studies included in this review exhibit significant heterogeneity in population characteristics, dosing regimens, and outcome measures, limiting the reliability of evidence synthesis. Existing studies on cancer-related depressive states generally have small sample sizes, and most are single-center studies. A systematic review (65) of postoperative depression in breast cancer patients reported high heterogeneity (I^2^ = 96%), suggesting substantial variability in effect sizes across studies. Additionally, the lack of standardization in depression assessment tools complicates cross-study comparisons. Evidence in special populations, including elderly patients, those with hepatic or renal impairment, and individuals with multiple comorbidities, is particularly limited.

##### 4.2.3.4 Insufficient cancer-specific evidence

Although esketamine has been approved for treatment-resistant depression in the general population, evidence for its application in cancer-related depressive states remains limited. Existing studies predominantly focus on perioperative depression, with a scarcity of evidence for depressive states in non-surgical cancer patients. Cancer patients often receive complex medication regimens, presenting potential drug-drug interaction risks.

## 5 Conclusion

### 5.1 From mechanism to clinical translation: future perspectives on esketamine in the management of cancer-related depressive states

#### 5.1.1 Biomarker-based prediction of esketamine efficacy and individualized treatment outcomes

Given the distinct clinical and biological profiles common to specific patient subgroups, precision medicine is now widely recognized as a transformative strategy for advancing disease prevention, diagnosis, and treatment. Individualized prediction models have been validated in psychiatry and have catalyzed the emergence of precision psychiatry as a distinct academic and clinical discipline (66). Despite growing scholarly interest and ongoing efforts by clinicians to adopt alternative strategies, routine clinical practice continues to rely predominantly on subjective clinical interviews rather than objective, neurobiologically grounded assessments; this reliance has contributed to a plateau in the improvement of clinical outcomes across major mental disorders in recent years (67). Recent breakthroughs in biomarker discovery are now driving a new generation of precision medicine trials in psychiatry, enabling more targeted and mechanism-informed interventions. Against this backdrop, leveraging biomarkers to predict and monitor esketamine’s efficacy in alleviating cancer-related depressive symptoms represents a promising frontier in its clinical translation.

### 5.2 Exploration of new drug delivery systems

#### 5.2.1 Current limitations

Several critical limitations must be acknowledged. First, clinical translation of nanocarrier systems faces barriers including stability, biocompatibility, and reproducibility (68). Second, the safety profile of nanocarriers, particularly carbon nanotubes, remains incompletely characterized (69). Third, no empirical studies have investigated esketamine-loaded nanocarriers for cancer-related depressive states. Fourth, key evaluation indicators such as targeting efficiency and brain penetration have not been established.

#### 5.2.2 Proposed research protocol and key evaluation indicators

Drawing on the targeted delivery strategy established by Gu et al. (70), the present review proposes a three-phase research protocol for the development of esketamine-loaded nanocarriers. Phase 1 involves nanocarrier preparation and characterization, with key indicators including particle size, drug loading capacity, and encapsulation efficiency. Phase 2 comprises *in vitro* and *in vivo* validation, evaluating release kinetics, cellular uptake, blood-brain barrier penetration, and efficacy in animal models of cancer-related depressive states, with key indicators including cumulative release profile, cellular uptake efficiency, behavioral outcomes such as tail suspension test and forced swim test, tumor volume, and plasma and brain levels of TNF-α, IL-6, and BDNF. Phase 3 focuses on safety assessment, evaluating body weight, liver and kidney function, and histopathology. For verification, independent replication in a second animal model is recommended before proceeding to clinical trials.

### 5.3 The potential value of “esketamine + immunotherapy”

#### 5.3.1 Current limitations

The combination of esketamine and immunotherapy lacks empirical support. Key limitations include no preclinical studies examining esketamine’s immunomodulatory effects in cancer immunotherapy, unexplored drug-drug interactions with immune checkpoint inhibitors, unknown optimal dosing and sequencing, and no validated predictive biomarkers (14).

#### 5.3.2 Proposed research protocol and key evaluation indicators

Based on existing evidence that esketamine possesses anti-inflammatory and immunomodulatory properties (71), we propose a stepwise research protocol. Preclinical proof-of-concept studies should first be conducted using tumor-bearing mouse models with chronic stress to evaluate esketamine alone and in combination with PD-1/PD-L1 inhibitors. Recent evidence demonstrates that esketamine can serve as an immune modulator by increasing mature dendritic cells and activated T cells while decreasing regulatory T cells in the tumor microenvironment (71).

For clinical translation, a randomized, double-blind, placebo-controlled phase II clinical trial should be designed, targeting cancer patients receiving PD-1/PD-L1 inhibitors with moderate-to-severe depressive symptoms defined as MADRS score of 20 or higher. The intervention would consist of intranasal esketamine 56 to 84 milligrams twice weekly for 4 weeks followed by weekly for 4 weeks, combined with maintenance immunotherapy. The primary endpoint is change in MADRS score from baseline to week 8, with secondary endpoints including objective response rate, progression-free survival, and quality of life. Exploratory biomarkers should include MDSC levels, M1/M2 ratio, serum BDNF, and plasma IL-6, TNF-α, and IL-1β (72). Verification pathways include independent validation cohorts, parallel mechanistic studies, and prospective biomarker qualification.

### 5.4 Summary and future perspectives

Esketamine has been widely adopted in anesthesia for cancer-related surgeries and demonstrates promising potential in ameliorating depressive symptoms in patients, underscoring its significant therapeutic value. However, as an emerging pharmacological agent, the mechanisms underlying its antidepressant effects remain incompletely understood, particularly regarding its efficacy in patients with mild cancer-related depressive states, and current administration strategies are largely limited to single-modal use. Therefore, future research should prioritize the following directions: (1) Elucidating the neurobiological mechanisms through preclinical studies using germ-free mice, transgenic mouse models, and non-human primates; (2) Conducting interdisciplinary clinical investigations in collaboration with oncology and psychiatry departments to evaluate esketamine’s efficacy in patients with mild cancer-related depressive states; (3) Employing integrated multi-omics approaches—including metagenomics, metabolomics, and transcriptomics—to comprehensively dissect the molecular pathways involved in its therapeutic action; and (4) Exploring combination regimens by leveraging established treatment protocols for psychiatric disorders, thereby optimizing synergistic effects and expanding the clinical utility of esketamine.

## Data Availability

The original contributions presented in the study are included in the article/supplementary material. Further inquiries can be directed to the corresponding authors.
